# Association of serum magnesium levels with risk factors, severity and prognosis in ischemic and hemorrhagic stroke patients

**DOI:** 10.22088/cjim.11.1.83

**Published:** 2020

**Authors:** Sadra Samavarchi Tehrani, Seyyed Hossein Khatami, Payam Saadat, Mohammad Sarfi, Alijan Ahmadi Ahangar, Roozbeh Daroie, Alireza Firouzjahi, Mahmood Maniati

**Affiliations:** 1 Department of Clinical Biochemistry, School of Medicine, Tehran University of Medical Sciences, Tehran, Iran; 2 Student Scientific Research Center, Tehran University of Medical Sciences, Tehran, Iran; 3 Department of Biochemistry, School of Medicine, Shiraz University of Medical Sciences, Shiraz, Iran; 4 Mobility Impairment Research Center, Health Research Institute, Babol University of Medical Sciences, Babol, Iran; 5 Student Scientific Research Center, Tehran University of Medical Sciences, Tehran, Iran; 6 Clinical Laboratory Section, Rouhani Hospital Babol University of Medical Sciences; 7 School of Medicine, Ahvaz Jundishapur University of Medical Sciences, Ahvaz, Iran

**Keywords:** Strokes, Magnesium, Ischemic stroke, Hemorrhagic stroke

## Abstract

**Background:**

Stroke is the third leading cause of mortality worldwide. One of the factors that affect the occurrence of stroke can be attributed to changes in the levels of trace elements. Accumulating evidence has been shown that magnesium, as an important element, is a new predictor of stroke. We aimed to determine the levels of Mg in ischemic stroke patients in comparison with those having the hemorrhagic type.

**Methods:**

This study was conducted on 447 stroke patients. Demographic characteristics of patients, stroke severity, and risk factors such as hypertension, ischemic heart disease, diabetes mellitus, and hyperlipidemia were recorded. Stroke was diagnosed based on the neurological examination and neuroimaging findings e.g. computed tomography (CT) or magnetic resonance imaging (MRI). The colorimetric technique was used to determine the concentration of Mg at 450 nm according to the commercial kit.

**Results:**

The mean of magnesium levels in ischemic patients was significantly higher than that in the hemorrhagic patients (P=0.001). Difference in magnesium status was associated with gender in thrombotic patients (P<0.05), while hyperlipidemia was associated with the status of magnesium in embolic patients (P=0.012). Furthermore, magnesium levels were correlated with ischemic heart disease in embolic (P=0.011) and sub-arachnoid hemorrhagic (SAH) patients (P=0.012), and with diabetes mellitus in thrombotic patients (P=0.012). Magnesium status was associated with the severity of ischemic stroke at the time of discharge in ischemic patients (P<0.001). Mg levels had the best area under curve (AUC) for the discrimination of ischemic patients from hemorrhagic ones.

**Conclusion:**

Magnesium levels were higher in ischemic patients compared to hemorrhagic ones, and these levels were associated with many risk factors contributing to a stroke. Magnesium may be used as a new predictor of stroke in ischemic patients as opposed to hemorrhagic ones.

Stroke is the leading cause of disability and mortality worldwide. Over 80% of all strokes in the world happen in the developing countries and studies conducted on these regions indicate remarkably higher fatality rates (1, 2). This neurological disorder imposes so many financial burdens on families and countries that it has become one of the most important healthcare issues in the world (3). According to the studies conducted in Iran, the incidence of stroke is 45 patients per 100,000 individuals among different populations (4). The main risk factors contributing to a stroke in Iran, like other parts of the world, include HTN (54%), hypercholesterolemia (15%), smoking (12%), IHD and DM (5).

Studies have demonstrated that stroke is associated with alterations in the balance of some trace elements. Indeed, trace elements are essential for maintaining the metabolism of neurons and glia (6, 7). Mg is an important trace element within the vascular system, and accumulating evidence has demonstrated that Mg has a key role in the pathogenesis of stroke and restoration of brain cells (8, 9). For example, animal studies have shown that Mg has a protective role in the integrity of blood brain barrier (BBB) and reduces edema after stroke as well as increases the antioxidant capacity in the lesion area through stimulation of antioxidant enzymes (10). The protective role of Mg at different age categories is discussed in details by Ingram Lingam and Nicola H Robertson in the review (11). It has also been shown that subjects with stroke have abnormalities in their serum and CNS Mg levels (12). Some studies indicate that a low serum Mg level promotes the risk of ischemic stroke, partially through affecting type 2 diabetes, HTN, and metabolic syndrome (13, 14) although some have suggested that Mg is independently associated with cerebrovascular diseases (15, 16). However, few, if any, studies have ever examined Mg status among ischemic and hemorrhagic stroke patients. Therefore, the purpose of this study was to investigate the serum level of Mg among ischemic and hemorrhagic stroke patients and its relationship with the risk factors of these subtypes of stroke such as HTN, IHD, DM, and HLP, as well as the severity of stroke at admission and discharge to find whether Mg status can be introduced as a novel predictor in these patients.

## Methods

**Study population: **This cross-sectional analytic study was performed on 447 stroke patients recruited from Ayatollah-Rouhani Hospital in Babol, Iran. This hospital is the main center for admission of stoke patients in Babol and the surrounding areas, and the patients of the present study were chosen from among those referring to the hospital from May 2015 to May 2017. Written consent was obtained from all subjects. The study was approved by the Ethics Committee of the Babol University of Medical Sciences (3407-9542120). Demographic characteristics of patients, type and severity of stroke, risk factors such as HTN, IHD, DM, HLP, and smoking were recorded in a checklist. The study population was divided into two main groups (ischemic stroke group and hemorrhagic stroke group), and each main group was further divided into two sub-groups, namely: embolic and thrombotic for the ischemic group, and intracerebral hemorrhagic (ICH) and sub-arachnoid hemorrhagic (SAH) for the hemorrhagic group. Stroke was diagnosed based on the neurological examination and neuroimaging findings. Computed tomography (CT) or magnetic resonance imaging (MRI) was performed for all patients. Although CT scans determine subarachnoid blood in patients with CT-negative, lumbar puncture (LP) was performed to determine subarachnoid blood(4). Diagnosis of stroke and its types was made according to the criteria followed in epidemiological studies of stroke (17). Definitive diagnosis of all stroke cases was confirmed by the neurologist responsible to do the project.

Severity of stroke was determined on the basis of NIH Stroke Scale (NIHSS) criteria, (18) where score ≤ 8 mild , 9 – 15 moderate and ≥ 16 severe stroke. HTN was defined as more than 140 mm-Hg for systolic blood pressure (SBP) or more than 90 mm-Hg for diastolic blood pressure (DBP). DM was defined as fasting blood sugar (FBS) more than 126 mg/dL, HLP as deregulated lipid profile patients including low level of HDL, high level of LDL and high triglyceride (19), and history of ischemic heart disease (IHD) was obtained from background information, history of cardiac medications, and ECG. Finally, patients were categorized as smokers if they had smoked at least 5 cigarettes per day in the last year.

**Exclusion criteria: **Exclusion criteria for the patients were cases with hemiparesis or any focal neurological findings other than stroke including head trauma, metabolic encephalopathy, brain abscess, brain tumor, migraine attacks, and seizure (19). Transient ischemic attack (TIA) cases were also excluded from the study. Besides, patients with renal failure, liver disorders, and those taking corticosteroid drugs were excluded.

**Blood sampling and laboratory assessment: **Serum samples were taken immediately after diagnosis of stroke and placed at -20˚C until use. Biochemical parameters including triglyceride (TG), FBS, and total cholesterol were measured using a commercially available kit. The serum Mg level was measured by Pars Azmoon kit (Pars Azmoon corporation, Iran, cat: 126500) according to the protocol. Magnesium was assessed based on xylidyl-blue which at alkaline ph Mg and xylidyl-blue produce a purple colored complex. The colorimetric technique was used to determine the concentration of Mg at 450 nm. The normal level of Mg was considered between 1.5 and 2.3 g/dl, and the values less than 1.5 g/dl were considered as low level while values more than 2.3 g/dl were considered as high level(19). The serum Mg levels were measured along with other laboratory tests in the clinical laboratory of Ayatollah-Rouhani Hospital in Babol.

**Statistical analysis: **In our study, the statistical data were analyzed quantitatively and qualitatively using SPSS (Version 24.0; SPSS Inc., Chicago, IL, USA). To determine the association of Mg serum levels with prognosis of stroke, we used chi-square test, and the relationship between other variables influencing the type of stroke and Mg serum status was examined by Fisher's Exact Test with calculation of odds ratio (OR) and 95% confidence interval (95% CI). Furthermore, p<0.05 were considered as significant. The diagnostic accuracies of Mg for the discrimination of ischemic stroke and hemorrhagic stroke patients were analyzed by receiver operating characteristic curves (ROC). The optimal cut-off values were selected based on the maximum value of sensitivity and specificity.

## Results

Included in this cross-sectional study were 447 stroke patients of whom, 225(50.3%) were males and 222 (49.7%) were females. As with the type of stroke, 374 had the ischemic type, of whom 170 patients were embolic and the rest were thrombotic while of the 73 having the hemorrhagic type, 26 were SAH and 47 were ICH. With regard to the relationship between stroke types and risk factors, we observed that there was a significant difference between HTN and the types of stroke and their subgroups (thrombotic/embolic, ICH/SAH) (P=0.026, 0.043 and 0.003, respectively). In addition, 29 patients having ICH stroke and 9 having SAH stroke had diabetes, while 18 having ICH as well as 17 having SAH did not have this disorder. Thus, according to this main risk factor, there was a significant difference between the subtypes of hemorrhagic stroke and DM (P=0.027). Moreover, considering the severity and status of stroke at discharge, we found a significant difference between the severity of stroke at the time of discharge in two types of stroke (P=0.034) and the subtypes of ischemic stroke (P=0.005). We also observed that 36.9% of the ischemic stroke patients and 76.7% of those having the hemorrhagic type had hypomagnesemia. Besides, the mean±SD serum levels of Mg in ischemic and hemorrhagic groups were 1.63±0.42 mg/dl, and 1.31±0.37 mg/dl respectively, and there was a significant difference between the two types of stroke with serum Mg status (P=0.001) while we did not find significant difference between serum Mg levels and both subtypes of ischemic as well as the hemorrhagic stroke (P=0.139, 0.426, respectively) (table 1).

The results of our data demonstrated that the only significant difference in serum levels of Mg was between both genders of thrombotic stroke patients (P=0.049). According to our findings, there was a significant association between the subtypes of ischemic stroke (i.e., thrombotic and embolic) and HTN, so in the thrombotic patients having a history of HTN as opposed to thrombotic patients not having a history of HTN, serum levels of Mg was low, and these findings were also observed for embolic patients (both of them P<0.001). 

Moreover, there was a significant association of serum Mg levels with severity of ischemic stroke in comparison with hemorrhagic stroke at the time of discharge (P>0.05), and we also found that serum Mg levels were significantly associated with the severity of ischemic stroke at the time of discharge in thrombotic (P<0.001) and embolic patients (P=0.003), but they were not significantly associated with severity at the time of discharge in the hemorrhagic group (P>0.05). 

Meanwhile, we did not find a significant association between serum status of Mg with severity of stroke at the time of admission in ischemic and hemorrhagic groups plus their sub-groups (P>0.05 all of them). Besides, there were significant relationships between serum Mg levels with DM only in thrombotic groups (P=0.012). Finally, of the 447 stroke cases, only in the subgroup of embolic, Mg serum status was associated with HLP (P=0.012) (table 2). We used logistic regression test to determine the effect of the measured variables on the risk of ischemic stroke. These findings demonstrated that serum Mg status is a risk factor for ischemic stroke and has a significant role (P=0.006, OR=5.43 and CI 95%= 1.61-18.31) (table 3).

In addition, to estimate the diagnostic accuracy of Mg for the distinction of ischemic stroke patients from those having hemorrhagic stroke, ROC analysis was performed and the results were presented in figure 1.  According to the ROC analysis, the cutoff value of 1.35 mg/dl serum levels of Mg showed the best diagnostic accuracy for discriminating ischemic stroke patients from hemorrhagic ones: AUC=0.71, P< 0.001 and 95% CI = (0.634 – 0.758).

**Table 1:** Frequency of stroke patients according to gender, severity of stroke and distribution of risk factors, as well as the mean±standard deviation (SD) serum levels of Mg in type of stroke and subtype of ischemic (embolic and thrombotic) as well as hemorrhagic of stroke (ICH and SAH)

		**Ischemic of stroke**			**Hemorrhagic of stroke**		
**Variable**	**Subgroup**	**Thrombotic ** **%(N)**	**Embolic** **%(N)**	P.value*	**ICH** **%(N)**	**SAH** **%(N)**	P.value**
Gender	male	57.4(109)	42.6(81)	0.261	68.6(24)	31.4(11)	0.473
	female	51.6(95)	48.4(89)		60.5(23)	39.5(15)	
HTN	Yes	60.5(98)	39.5(64)	0.043	78.6(33)	21.4(9)	0.003
	NO	50(106)	50(106)		45.2(14)	54.8(17)	
IHD	Yes	50(81)	50(81)	0.123	64.9(24)	35.1(13)	0.931
	NO	58(123)	42(89)		63.9(23)	36.1(13)	
HLP	Yes	56.5(14)	43.5(170)	0.45	62.1(18)	37.9(11)	0.737
	NO	52.6(100)	47.4(90)		65.9(29)	34.1(15)	
DM	Yes	57.1(96)	42.9(72)	0.362	76.3(29)	23.7(9)	0.027
	NO	52.4(108)	47.6(98)		51.4(18)	48.6(17)	
Smoking	Yes	50.3(73)	49.7(72)	0.194	63.6(21)	36.4(12)	0.904
	NO	57.2(131)	42.8(98)		65.0(26)	35.0(14)	
Severity in admission time	Mild	54.1(146)	45.9(124)	0.348	68.0(34)	32.0(16)	0.424
	Moderate	52.4(44)	47.6(40)		60.0(12)	40.0(8)	
	Severe	70.0(14)	30.0(6)		33.3(1)	66.7(2)	
Severity in discharge time	Mild	47.6(89)	52.4(98)	0.034	63.0(17)	37.0(10)	0.632
	Moderate	60.7(65)	39.3(42)		72.2(13)	27.8(5)	
	Severe	58.6(34)	41.4(24)		68.8(11)	31.3(5)	
	death	72.7(16)	27.3(6)		50.0(6)	50.0(6)	
Mg (mg/dl)		Mean±SD	Mean±SD	0.139	Mean±SD	Mean±SD	
		1.60±0.44	1.67±0.40		1.28±0.38	1.36±0.41	0.426

* HTN: hypertension, IHD: ischemic heart disease, HLP: hyperlipidemia, DM: Diabetes Mellitus.*

P.value *: indicating significant difference between thrombotic and embolic patients in ischemic of stroke.

P.value **: indicating significant difference between ICH and SAH patients in hemorrhagic of stroke.

**Table 2:** Stroke types, risk factors and severity, considering of serum levels of Mg

**Variable**			**Serum levels of Mg (%)**		**P.value**
**Type of stroke**			**Low**	**Normal**	
Ischemic	thrombotic	Female	31.6(30)	68.4(65)	0.049*
		male	45.0(49)	55.0(60)	
	Embolic	Female	33.7(70)	66.3(59)	0.774
		male	35.8(29)	64.2(52)	
Hemorrhagic	ICH	Female	73.9(17)	26.1(6)	0.231
		male	80.9(38)	19.1(9)	
	SAH	Female	60.0(9)	40.0(6)	0.234
		male	81.8(9)	18.2(2)	
		HLP			
Ischemic	thrombotic	Negative	36.0(36)	64.0(64)	0.433
		positive	41.3(43)	58.7(61)	
	Embolic	negative	43.3(56.7)	56.7(51)	0.012*
		positive	25.0(20)	75.0(60)	
Hemorrhagic	ICH	negative	86.2(25)	13.8(4)	0.236
		positive	72.2(13)	27.8(5)	
	SAH	negative	66.7(10)	33.3(5)	0.741
		positive	72.7(8)	27.3(3)	
		HTN			
Ischemic	thrombotic	Negative	25.5(27)	74.5(79)	<0.001*
		positive	53.1(52)	46.9(49)	
	Embolic	negative	21.7(23)	78.3(83)	<0.001*
		positive	56.3(36)	43.8(28)	
Hemorrhagic	ICH	negative	64.3(9)	35.7(5)	0.060
		positive	87.9(29)	12.1(4)	
	SAH	negative	70.6(12)	29.4(5)	0.837
		positive	66.7(6)	33.3(3)	
		Severity in discharge time			
Ischemic	thrombotic	Mild	24.7(22)	75.3(67)	<0.001*
		Moderate	40.0(26)	60.0(39)	
		Sever	73.5(25)	26.5(9)	
		death	37.5(6)	62.5(10)	
	Embolic	Mild	24.5(24)	56.7(51)	0.003*
		Moderate	40.5(17)	75.0(60)	
		Sever	58.3(14)	41.7(10)	
		death	66.7(4)	33.3(2)	
Hemorrhagic	ICH	Mild	64.7(11)	35.5(6)	0.145
		Moderate	92.3(12)	7.7(1)	
		Sever	81.8(9)	18.2(2)	
		death	100.0(6)	0.0(0)	
	SAH	Mild	40.0(4)	60.0(6)	0.07
		Moderate	80.0(4)	20.0(1)	
		Sever	100.0(5)	0.0(0)	
		death	83.3(5)	16.7(1)	
		IHD			
Ischemic	Thrombotic	Negative	35.0(43)	65.0(80)	0.174
		positive	25.8(23)	55.6(45)	
	Embolic	negative	25.8(23)	74.2(66)	0.011*
		positive	44.4(36)	55.6(45)	
Hemorrhagic	ICH	negative	69.6(6)	30.4(7)	0.060
		positive	91.7(23)	8.3(2)	
	SAH	negative	46.2(6)	53.8(7)	0.012*
		positive	92.3(12)	7.7(1)	
		DM			
Ischemic	Thrombotic	Negative	30.6(33)	69.4(75)	0.012*
		positive	47.9(46)	52.1(50)	
	Embolic	negative	32.7(33)	67.3(66)	0.512
		positive	37.5(27)	62.5(45)	
Hemorrhagic	ICH	negative	83.8(15)	16.7(3)	0.733
		positive	79.3(23)	20.7(6)	
	SAH	negative	76.5(13)	23.5(4)	0.272
		positive	55.6(5)	44.4(4)	
		Severity in admission time			
Ischemic	thrombotic	Mild	34.2(50)	65.8(96)	0.094
		Moderate	47.4(21)	52.3(23)	
		severe	26.6(8)	18.4(6)	
	Embolic	Mild	32.2(40)	67.7(84)	0.205
		Moderate	37.5(15)	62.5(5)	
		severe	66.7(4)	33.3(2)	
Hemorrhagic	ICH	Mild	85.3(29)	14.7(5)	0.237
		Moderate	75.0(9)	25.0(3)	
		severe	0(0)	100(1)	
	SAH	Mild	56.3(9)	14.7(5)	0.234
		Moderate	87.5(7)	12.5(1)	
		severe	100(2)	0(0)	

Intracerebral hemorrhage (*ICH*), *Subarachnoid hemorrhage* (*SAH*), Hyperlipidemia (HLP), Hypertension(HTN), Ischemic heart disease(IHD), Diabetes mellitus (DM). P.value*: indicating significant difference between groups (<0.05).

**Table 3:** The risk of ischemic stroke with adjusted variables by logistic regression test

**Variable **	**Odds Ratio (OR)**	**Confidence interval 95%**	**P.value**
Gender	0.85	0.28-2.36	0.79
Mg	5.43	1.61-18.31	0.006
Mild stroke severity at admission	1.00	-	0.43
Moderate stroke severity at admission	0.98	0.35-2.69	0.96
Severe stroke severity at admission	5.15	0.41-63.8	0.20
Mild stroke severity at discharge	1.00	-	0.47
Moderate stroke severity at discharge	0.58	0.16-2.16	0.42
Severe stroke severity at discharge	1.56	0.52-4.67	0.43
Death stroke severity at discharge	2.08	0.30-14.51	0.45
HTN	0.82	0.29-2.36	0.71
IHD	0.95	0.38-2.36	0.91
DM	1.29	0.52-3.19	0.57
HLP	0.59	0.22-1.56	0.29
Smoking	2.60	0.86-7.86	0.09

## Discussion

To the best of our knowledge, this is the first study to investigate the relationship between the serum levels of Mg and demographic features, risk factors, and severity among ischemic and hemorrhagic stroke patients. The results of our  study demonstrated that there was a significant difference between the Mg serum level detected in the ischemic stroke group and that of the hemorrhagic stroke patients. In contrast to our study, Bayir et al. reported that mean Mg serum status did not have a significant difference in ischemic and hemorrhagic stroke patients in comparison with a healthy group (P=0.74), which seems to be due to the small sample size (20). Similarly, Koksaldi et al. indicated that in acute stroke patients, the Mg serum levels did not have any statistically significant difference with those of the healthy group, and there was only a significant correlation between serum and CSF Mg levels (21). Additionally, another study reported that the mean serum Mg level in 200 patients with acute ischemic stroke was 1.71±0.51mg/dl of whom 32% had hypomagnesemia(22), while the mean Mg serum level in our study was 1.58±0.43 mg/dl of whom 37% had low Mg. Furthermore, Karadas et al. found that serum Mg levels were significantly lower in 26 patients with acute hemorrhagic stroke compared with 29 healthy individuals(23). However, our data showed that the mean of Mg levels in ischemic patients were higher than that of the hemorrhagic ones. These discrepancies between the studies mentioned may be due to geography of the living place, lifestyles and diet differences, which can significantly affect the results. However, the difference between the mean of Mg levels in different types of stroke may have a physiopathologic basis, which may be considered for future studies. 

Besides, we did not find any significant difference in serum Mg status between the subgroups of ischemic stroke (i.e., thrombotic and embolic) and those of hemorrhagic stroke (i.e., ICH and SAH). Furthermore, in a meta-analysis study, a modest but statistically significant inverse association between Mg intake and risk of stroke was observed. Daily intake increment of 100 mg Mg/d was associated with an 8% reduction in the risk of total stroke, and Mg intake was inversely associated with risk of ischemic stroke but not with ICH and SAH (14), which was consistent with our results.

Our findings also showed that the differences in Mg levels were significantly associated with gender in thrombotic patients and that HLP was significantly associated with Mg serum status in embolic patients. In this line, Akarolo-Anthony et al. observed that among women with ischemic stroke, the median status of Mg was not different in comparison with healthy individuals. They showed that there was no significant association between plasma level of Mg and the risk of total ischemic stroke, and when adjusted for HbA1c, history of DM, history of HTN, coronary heart disease, total/HDL cholesterol did not alter the risk estimates (RR=1.34, P.trend= 0.19)(24). In addition, Ohira et al. in a cohort study observed that status of serum Mg positively correlated with both LDL- and HDL-cholesterol levels in ischemic stroke patients. Furthermore, higher serum Mg levels were associated with lower prevalence of HTN and DM at baseline, and baseline serum Mg was inversely correlated with blood pressure and the prevalence of HTN and DM(13). Recently, a study reported that acute ischemic stroke patients with lower Mg levels also differed in lipid profile (higher HDL-cholesterol levels, but lower TG levels, and LDL-cholesterol levels), and that a significant correlation between Mg status at admission and gender and risk factors such as cigarette smoking, history of HTN, DM, coronary heart disease(25). HTN and DM could be mediators between serum Mg and the incidence of different types of stroke. Besides, these associations have also been detected in other prospective studies (26-28). Thus, the results of our study were similar to the studies of Ohira et al. and Shoujiang You et al.

Difference in Mg levels was significantly associated with HTN in both subgroups of ischemic stroke, but not with both subtypes of hemorrhagic stroke. However, according to the findings in DM patients, we observed that there was a significant association between the status of Mg and the presence or absence of DM only in thrombotic patients, as opposed to other subgroups of stroke. With respect to the results of a previous study that reported the Mg levels to be associated with DM and HTN in ischemic stroke patients(29, 30), we illustrated that Mg levels had a significant association with HTN in both subgroups of ischemic stroke and it was correlated with DM only in one subgroup of ischemic stroke (i.e., thrombotic patients). Given this finding, hypomagnesemia should be taken into account considering the relationship between serum Mg levels and DM and HTN in ischemic stroke patients, and this could be a subject for future studies.

Several studies have investigated the effect of baseline Mg on stroke outcome. The role of serum Mg levels in ischemic recovery, however, remains controversial and deserves further investigation(31, 32). Severity of stroke at discharge is an important prognostic factor, and we found in both subgroups of ischemic stroke that there was a significant association between Mg serum levels and severity at discharge, but not at admission. Currently, there are few studies conducted in this line of inquiry. However, Siegler et al. reported that there were no significant differences in severity of stroke between patients with low admission Mg relative to patients with normal-to-high serum Mg levels, and it was proposed that Mg groups at baseline were not predictive of poor functional outcome, death or discharge disposition(33). Therefore, future studies are needed to shed more light on the association between Mg and severity at admission and discharge times. If the relationship between hypomagnesemia and stroke severity is confirmed, the question arises whether hypomagnesemia leads to severe stroke or more severe stroke will lead to hypomagnesemia.

A more recent study has found that the plasma status of Mg was negatively associated with the risk of cardiovascular outcomes including coronary heart disease and atrial fibrillation (34, 35). Additionally, it appeared that serum Mg level was inversely correlated with von Willebrand factor status, which in turn was positively associated with the incidence of ischemic stroke (36). Hence, our findings indicate that serum Mg levels had a significant association with IHD in embolic ischemic stroke and in SAH of hemorrhagic stroke, which is similar to the results of the mentioned studies.

Considering the findings about the relationship between stroke types and its risk factors, we found that there was a significant difference between HTN with the type of stroke and its subgroups, and that DM as the main risk factor of stroke, had a significant difference with regard to the subtypes of hemorrhagic stroke. Moreover, there was a significant difference in the severity of stroke at discharge in terms of the two types of stroke and the subtypes of ischemic stroke. In this line, many studies have demonstrated that stroke and its subgroups are associated with some risk factors such as HTN, DM, IHD, and HLP (24, 25, 33), while our data did not show a significant association between IHD and HLP with types of stroke and their subgroups. In this regard, Ahangar et al. reported that hypertension was significantly associated with hemorrhagic stroke, while dyslipidemia was more associated with ischemic stroke. Meanwhile, they found that stroke was more prevalent in females (2). However, in the present study, from the risk factors studied, the HTN similarly was significantly associated with various types of stroke, while HLP, gender, IHD and smoking did not have significant association with the different types of stroke. 

According to the ROC analysis, we determined the diagnostic accuracy of the measured Mg and extracted the related cut-points and data. Based on the results of the ROC curve analysis, the cutoff value of 1.35 mg/dl serum levels of Mg showed the best diagnostic accuracy for discriminating ischemic stroke patients from hemorrhagic ones with the AUC value of=0.71 which makes Mg consider as a potential marker. Definitely evaluating the diagnostic accuracy of Mg in large and longitudinal studies can open a new window for possible more applications of these molecules in the clinic. However, further large scale studies are needed to verify our results, and there are still some potential limitations that merit considerations. For instance, our study did not have healthy individuals as a control group to compare patients with different types of stroke with healthy persons. Furthermore, the short follow-up time is another limitation. Despite these limitations, the relatively high number of studied patients in comparison with similar studies on this topic was one of the strengths of this study.

The mean of Mg levels in ischemic patients was significantly higher than hemorrhagic type. Difference in Mg levels was associated with gender in thrombotic patients, and HLP was associated with serum status of Mg in embolic patients. Besides, serum Mg levels were correlated with IHD in embolic and SAH patients and DM only in thrombotic patients. Moreover, serum Mg status was associated with the severity of ischemic stroke at discharge in ischemic stroke patients. Mg levels had the best AUC, sensitivity and specificity for discrimination of ischemic patients from hemorrhagic ones.
